# Vieussens’ arterial ring forming a coronary–pulmonary artery fistula with large aneurysmal dilatation: a case report

**DOI:** 10.1093/ehjcr/ytae507

**Published:** 2024-09-14

**Authors:** Shuji Moriyama, Kosuke Nakata, Hideki Doi, Toshiyuki Matsumura, Toshihiro Fukui

**Affiliations:** Department of Cardiovascular Surgery, Kumamoto Rosai Hospital, Yatsushiro, Kumamoto, Japan; Department of Cardiovascular Surgery, Kumamoto Rosai Hospital, Yatsushiro, Kumamoto, Japan; Department of Cardiovascular Medicine, Kumamoto Rosai Hospital, Yatsushiro, Kumamoto, Japan; Department of Cardiovascular Medicine, Kumamoto Rosai Hospital, Yatsushiro, Kumamoto, Japan; Department of Cardiovascular Surgery, Graduate School of Medical Sciences, Kumamoto University Hospital, Kumamoto, Japan

**Keywords:** Vieussens’ arterial ring, Coronary–pulmonary artery fistula, Coronary artery aneurysm, Case report

## Abstract

**Background:**

Vieussens’ arterial ring (VAR) is an embryonic conotruncal ring remnant that represents a rare vascular anomaly involving a connection between the right coronary artery (RCA) and the left anterior descending artery (LAD). We describe a unique case of VAR associated with large aneurysmal dilatation, which presents as the formation of a fistula between the coronary artery and pulmonary artery.

**Case summary:**

An 80-year-old Japanese woman presented with an asymptomatic mediastinal mass that was incidentally detected on computed tomography. Subsequent imaging over 2 years revealed the progression of the two masses connecting the RCA to the LAD measuring 8 × 7 mm and 28 × 21 mm in diameter. Transthoracic echocardiography identified a cystic lesion anterior to the right ventricular outflow tract, and colour Doppler imaging confirmed flow into the pulmonary artery. Furthermore, coronary angiography revealed a large aneurysm arising from the LAD, with an efferent vessel communicating with the pulmonary artery. Surgical intervention involved resection of the aneurysms and closure of the coronary–pulmonary artery fistula, which yielded favourable postoperative outcomes.

**Discussion:**

Vieussens’ arterial ring is a rare but clinically significant anomaly with varied presentations; the incidence of VAR remains uncertain. In this case, surgical resection and closure of the fistula were performed to mitigate the risk of rupture and to address the potential haemodynamic consequences. Understanding and documenting such cases will contribute to refining treatment approaches and improving patient care in cardiovascular medicine.

**Learning points**

- Vieussens’ arterial ring (VAR) is an embryonic conotruncal ring remnant that represents a rare vascular anomaly involving a connection between the right coronary artery (RCA) and the left anterior descending artery (LAD). Vieussens’ arterial ring provides collateral blood flow to the myocardium distal to the occlusion, reducing ischaemia and protecting the myocardium from infarction.
- The main symptom of VAR is angina associated with coronary artery disease, coronary artery aneurysm, or coronary artery fistula. Vieussens’ arterial ring presenting with both aneurysmal formation and a coronary artery-to-pulmonary artery fistula is rare.
- There is no consensus on the optimal management of these patients, and treatment decisions should be tailored to each patient. We adopted a surgical procedure after collaborative evaluation by our heart team, considering the patient’s will.

## Introduction

Vieussens’ arterial ring (VAR) is an embryonic conotruncal ring remnant that refers to the connection between the conus branch of the right coronary artery (RCA) and the proximal right ventricular branch of the left anterior descending artery (LAD).^1,2^ Vieussens’ arterial ring provides collateral blood flow to the myocardium distal to the occlusion, reducing ischaemia and protecting the myocardium from infarction. Although the incidence of VAR is unknown, VAR presenting with both aneurysmal formation and a coronary artery-to-pulmonary artery fistula (CPAF) is rare.^1^ Although several similar cases have been reported,^3–7^ no established guidelines exist for the treatment of these diseases. Thus, the management plan is determined based on individual symptoms or the state of the patients. We report a rare case of VAR forming a CPAF with large aneurysmal dilatation managed by surgical intervention.

## Summary figure

**Figure:**
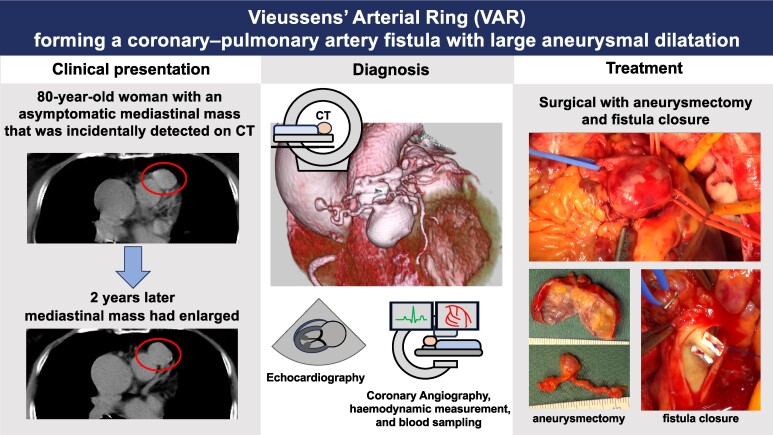


## Case presentation

**Figure 1:**
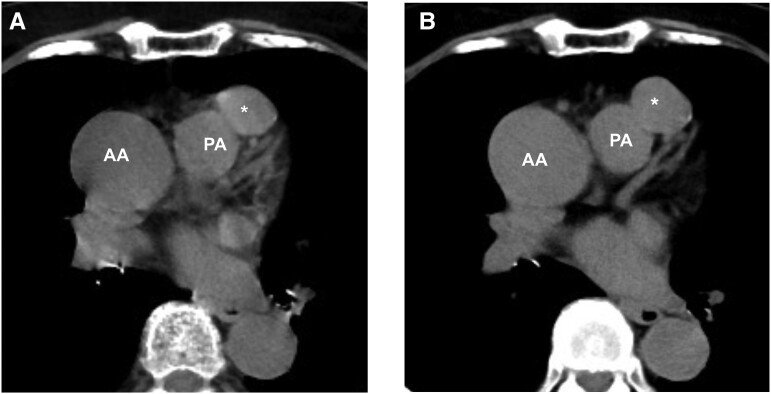
Preoperative computed tomography 2 years prior (*A*) and at referral to our hospital (*B*). The asterisk indicates a large aneurysm. AA, ascending aorta; PA, main pulmonary artery.

An 80-year-old Japanese woman with an asymptomatic mediastinal mass that was incidentally detected by computed tomography (CT) was referred to our hospital. Chest auscultation revealed no cardiac murmur. The mediastinal mass was first detected on plain CT 2 years prior; since then, the size of the mass had gradually enlarged (*Figure 1*). She did not present with any angina-like symptoms on exertion or ischaemic changes on electrocardiography.

**Figure 2:**
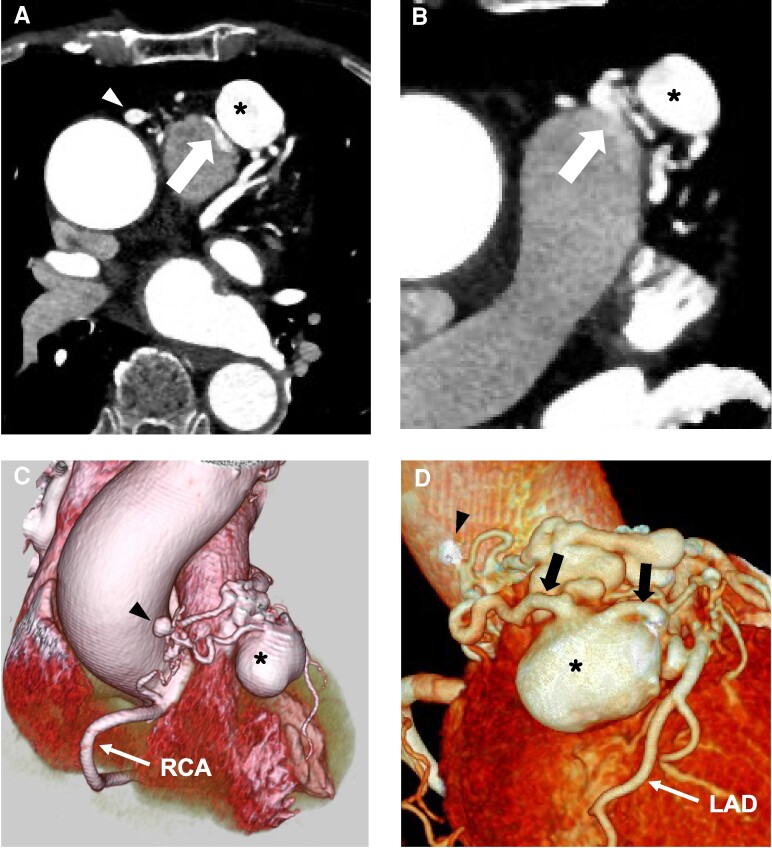
Preoperative multidetector computed tomography. The asterisk indicates a large aneurysm. Computed tomography coronary angiography axial view (*A* and *B*) showing a small aneurysm (arrowhead) and coronary artery-to-pulmonary artery fistula (arrow). Heart surface volume rendering reconstructions (C and *D*) showing a small aneurysm (arrowhead) and large aneurysm (*) with the afferent and efferent vessels (arrows) connecting the left anterior descending artery and right coronary artery.

**Figure 3:**
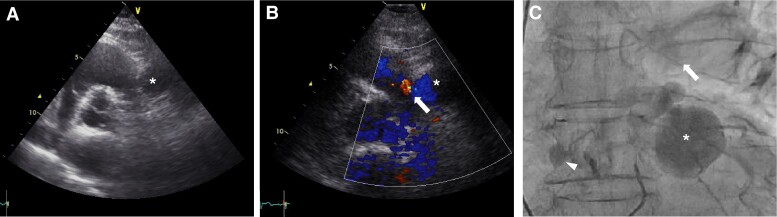
Transthoracic echocardiography showing a round cystic lesion (*) located in front of the right ventricular outflow tract (*A*) and efferent flow into the pulmonary artery (arrow) on colour Doppler flow imaging (*B*). Coronary angiography of the left coronary artery shows both a small aneurysm of the right coronary artery (arrowhead) and the coronary pulmonary artery fistula connecting the proximal branch of the left anterior descending artery to the main pulmonary artery (arrow) through the large aneurysm (*) (*C*).

The mediastinal mass detected on plain CT was suspected to be related to the coronary arteries; therefore, an initial contrast-enhanced CT was performed. Multidetector CT (MDCT) revealed a small mass and a large mass measuring 8 × 7 mm and 28 × 21 mm in diameter, respectively, connecting the conus branch of the RCA and the LAD (*Figure 2*; Supplementary material online, *Video S1*). Transthoracic echocardiography (TTE) revealed a round cystic lesion located in front of the right ventricular outflow tract (*Figure 3A*), and efferent flow into the pulmonary artery was observed with colour Doppler flow imaging (*Figure 3B*; Supplementary material online, *Video S2*). Furthermore, coronary angiography revealed a large aneurysm arising from the proximal branch of the LAD and efferent vessel to the pulmonary artery (*Figure 3C*; Supplementary material online, *Video S3*). Oximetry revealed an oxygen step-up of 4% between the pulmonary artery and right ventricle, which is consistent with a left-to-right shunt of 5.0%. The pulmonary-to-systemic blood flow ratio (Qp/Qs) was 1.18. Therefore, based on these findings, the patient was diagnosed with a CPAF with a concomitant large aneurysm that was considered to have progressively grown over 2 years.

We discussed the risks of rupture of a CPFA with large coronary artery aneurysms and the benefits of possible therapeutic options, such as medical therapy, percutaneous coronary intervention, and surgical treatment, with the heart team of our hospital. Although the patient was 80 years old and asymptomatic, she was judged to be operationally tolerant. The management plan was determined in accordance with the patient’s and her family’s will. The findings were discussed with the patient and her family members, the risks and benefits were clarified, and the patient expressed a preference for open-heart surgery.

**Figure 4.**
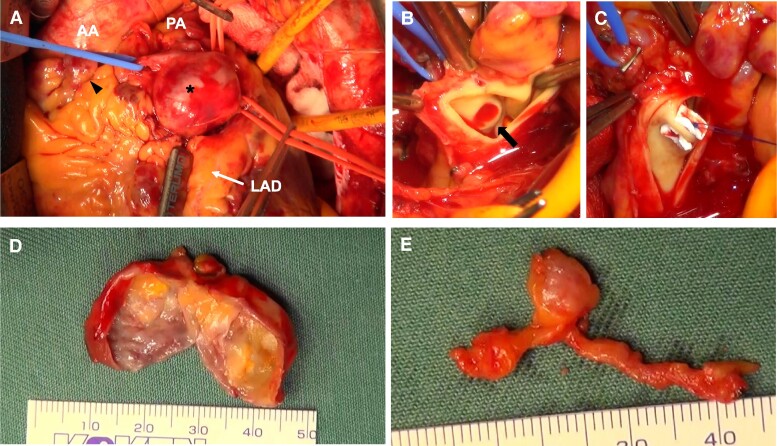
Intraoperative findings (*A*, *B*, and *C*) and resected aneurysms (*D* and *E*). Asterisk indicating the large aneurysm. The arrowhead indicates the small aneurysm (*A*). The arrow indicates the coronary pulmonary fistula (*B*). AA, ascending aorta; PA, main pulmonary artery; LAD, left anterior descending artery.

We subsequently performed coronary aneurysm resection and CPAF closure. A large and a small cardiac mass, with a maximum diameter of ∼30 and 10 mm, respectively, was found in front of the right ventricular outflow tract connecting the branch of the RCA and the LAD (*Figure 4A*). The afferent vessels were confirmed to be connected to the LAD and RCA. Under cardiac arrest using cardiopulmonary bypass, the aneurysms were resected after the afferent and efferent vessels were individually ligated (*Figures 4D* and *E*). There was a hole slightly <10 mm in diameter in contact with the main pulmonary artery (*Figure 4B*). The afferent vessel was ligated to the pulmonary artery. Additionally, the CPAF was directly closed on the luminal side of the pulmonary artery (*Figure 4C*).

Histologically, the aneurysm wall retained a three-layered structure, and no atherosclerotic changes were observed. Postoperative MDCT and TTE revealed no evidence of a coronary artery aneurysm or abnormal shunt flow into the pulmonary artery.

## Discussion

In 1706, Raymond de Vieussens (1641–1715), a French physician and anatomist, noted several important new cardiac findings, including a vascular connection of the conus branch of the RCA circling around the aorta to the left coronary arterial system.^8^ This collateral system connects the RCA to the LAD, which allows preserved flow despite significant obstruction of either vessel. Although the incidence rate of VAR is unknown, Doğan *et al*. reported that the detection rate of VAR was 0.319% when assessing 3443 consecutive coronary CT angiography examinations.^1^ The main symptoms are angina associated with coronary artery disease, coronary artery aneurysm, or CPAF.^2^ Furthermore, VAR with both aneurysmal formation and CPAF is rare.^1^

The incidence of coronary artery aneurysms ranges from 0.3% to 5.3%.^9^ Although coronary artery aneurysms are not uncommon, large coronary artery aneurysms with a diameter > 20 mm are rare and have been reported to occur in only 0.02% of patients.^10^ There is no consensus on the optimal management of coronary artery aneurysms, and treatment decisions should be tailored to each patient. Generally, a coronary artery aneurysm > 30 mm is an indication for surgery due to the risk of rupture.^7^

Furthermore, CPAF is also a rare condition, with a prevalence ranging from 0.17% to 0.68%.^11,12^ The indications for interventions in CPAF are controversial. Although a Qp/Qs > 1.5 is one indication for repair of left-to-right shunt disease, such as an atrial septal defect,^13,14^ there is no definitive indication for interventions in CPAF. According to the guidelines for the management of adult congenital heart disease, medium- or large-sized CPFA should be closed regardless of the symptomatology; in contrast, a small-sized fistula should be closed in patients who are symptomatic with myocardial ischaemia, arrhythmias, ventricular dilatation, or dysfunction of uncertain origin, or if the fistula is complicated by endocarditis.^13,14^

A combination of coronary artery aneurysm and CPAF is extremely rare. We report a rare case of VAR forming a CPAF with large coronary artery aneurysmal dilatation.

The risk of rupture of a CPAF with an aneurysm may be greater than that of a native coronary artery aneurysm due to the fragile structure of the fistula.^7^ Resection of the aneurysms after ligation of both the afferent and efferent vessels and direct closure of the CPAF at the luminal side of the pulmonary artery were performed.

In conclusion, we present a rare case of VAR forming a CPAF with a large aneurysmal dilatation. Management of such complex cases requires careful consideration and tailored approaches. In the present case, aneurysm resection and fistula closure were performed to mitigate the risk of rupture and alleviate any potential haemodynamic consequences. This case underscores the importance of recognizing and appropriately managing rare cardiovascular anomalies to ensure an optimal patient outcome.

## Supplementary Material

ytae507_Supplementary_Data

## Data Availability

No new data were generated or analysed in support of this article.

## Lead author biography

Dr Shuji Moriyama is working in the Department of Cardiovascular Surgery at Kumamoto Rosai Hospital in Yatsushiro, Japan, specializing in cardiovascular surgery, especially in valvular heart disease, coronary disease, and aortic disease.

## Notes

**Consent:** The authors confirm that written consent for the submission and publication of this case report, including images and associated text, was obtained from the patient, in line with the COPE guidelines.

**Funding:** None declared.

## References

[1] Doğan N, Dursun A, Özkan H. Vieussens’ arterial ring: a rare coronary variant anatomy. Diagn Interv Radiol 2019;25:109–113. 30860074 10.5152/dir.2019.17449 PMC6411272

[2] Christodoulou KC, Stakos D, Androutsopoulou V, Chourmouzi-Papadopoulou M, Tsoucalas G, Karangelis D, et al Vieussens’ arterial ring: historical background, medical review and novel anatomical classification. Cureus 2023;15:e40960. 37378305 10.7759/cureus.40960 PMC10291275

[3] Yoshihara S, Yaegashi T, Matsunaga M, Kurata M, Naito M. Multimodality imaging in a case of coronary to pulmonary artery fistula with multiple aneurysms via a Vieussens arterial ring. Circ Cardiovasc Imaging 2021;14:e012178. 34034503 10.1161/CIRCIMAGING.120.012178

[4] Pujitha V, Pandey NN, Deepti S, Kumar S. Incidentally detected Vieussens’ arterial ring-to-pulmonary trunk fistula in a patient with aortic stenosis. J Card Surg 2022;37:3882–3883. 35924997 10.1111/jocs.16828

[5] Hagiwara H, Takahashi A, Komoriyama H, Kato Y, Anzai T. Vieussens’ arterial ring forming a fistula that drains into the pulmonary artery through an aneurysm. Eur Heart J Cardiovasc Imaging 2023;24:e104. 37013987 10.1093/ehjci/jead048

[6] Takahashi T, Wakatsuki T, Ise T, Sata M. Spontaneous thrombosis of a giant aneurysm complicated with the coronary-to-pulmonary artery fistula: a case report. Eur Heart J Case Rep 2024;8:ytae227. 38736999 10.1093/ehjcr/ytae227 PMC11087926

[7] Shibata K, Maeda S, Kawamura M, Nakatsuji H, Ryugo M, Tsutsumi Y, et al Successful surgical treatment for ruptured aneurysm of coronary-pulmonary artery fistula complicated with cardiac tamponade. J Am Coll Cardiol Case Rep 2022;4:1283–1287. 10.1016/j.jaccas.2022.08.026 PMC9666919 36406920

[8] Parker J . Raymond de Vieussens (1641–1715) French neuroanatomist and physician. JAMA 1968;206:1785–1786. 4881371

[9] Abou Sherif S, Ozen Tok O, Taşköylü Ö, Goktekin O, Kilic ID. Coronary artery aneurysms: a review of the epidemiology, pathophysiology, diagnosis, and treatment. Front Cardiovasc Med 2017;4:24. 28529940 10.3389/fcvm.2017.00024 PMC5418231

[10] Pham V, Hemptinne Q, Grinda JM, Duboc D, Varenne O, Picard F. Giant coronary aneurysms, from diagnosis to treatment: a literature review. Arch Cardiovasc Dis 2020;113:59–69. 31866173 10.1016/j.acvd.2019.10.008

[11] Kim H, Beck KS, Choe YH, Jung JI. Coronary-to-pulmonary artery fistula in adults: natural history and management strategies. Korean J Radiol 2019;20:1491–1497. 31606954 10.3348/kjr.2019.0331 PMC6791815

[12] An X, Guo S, Dong H, Tang Y, Li L, Duan X, et al Congenital coronary artery-to-pulmonary fistula with giant aneurysmal dilatation and thrombus formation: a case report and review of literature. BMC Cardiovasc Disord 2021;21:273. 34088261 10.1186/s12872-021-02077-4 PMC8176730

[13] Stout KK, Daniels CJ, Aboulhosn JA, Bozkurt B, Broberg CS, Colman JM, et al 2018 ACC/AHA guidelines for the management of adults with congenital heart disease: a report of the American College of Cardiology/American Heart Association Task Force on Practice Guidelines. Circulation 2019;139:e698–e800. 30586767 10.1161/CIR.0000000000000603

[14] Baumgartner H, De Backer J, Bavu-Narayan SV, Budts W, Chessa M, Diller GP, et al 2020 ESC guidelines for the management of adult congenital heart disease: the task force for the management of adult congenital heart disease of the European Society of Cardiology (ESC). endorsed by: Association for European Paediatric and Congenital Cardiology (AEPC), International Society for Adult Congenital Heart Disease (ISACHD). Eur Heart J 2021;42:563–645. 32860028 10.1093/eurheartj/ehaa554

